# Impaired Subcortical Processing of Amplitude-Modulated Tones in Mice Deficient for *Cacna2d3*, a Risk Gene for Autism Spectrum Disorders in Humans

**DOI:** 10.1523/ENEURO.0118-22.2022

**Published:** 2022-04-21

**Authors:** Gerhard Bracic, Katrin Hegmann, Jutta Engel, Simone Kurt

**Affiliations:** 1 School of Medicine, Department of Biophysics, Center for Integrative Physiology and Molecular Medicine (CIPMM), Saarland University, Homburg 66421, Germany; 2 Institute of Neurobiology, University of Ulm, Ulm 89069, Germany

**Keywords:** auditory processing disorder, autism spectrum disorders, Ca^2+^ channel subunit, *Cacna2d3*, inferior colliculus, temporal coding

## Abstract

Temporal processing of complex sounds is a fundamental and complex task in hearing and a prerequisite for processing and understanding vocalization, speech, and prosody. Here, we studied response properties of neurons in the inferior colliculus (IC) in mice lacking *Cacna2d3*, a risk gene for autism spectrum disorders (ASDs). The α_2_δ3 auxiliary Ca^2+^ channel subunit encoded by *Cacna2d3* is essential for proper function of glutamatergic synapses in the auditory brainstem. Recent evidence has shown that much of auditory feature extraction is performed in the auditory brainstem and IC, including processing of amplitude modulation (AM). We determined both spectral and temporal properties of single- and multi-unit responses in the IC of anesthetized mice. IC units of α_2_δ3^−/−^ mice showed normal tuning properties yet increased spontaneous rates compared with α_2_δ3^+/+^. When stimulated with AM tones, α_2_δ3^−/−^ units exhibited less precise temporal coding and reduced evoked rates to higher modulation frequencies (f_m_). Whereas first spike latencies (FSLs) were increased for only few modulation frequencies, population peak latencies were increased for f_m_ ranging from 20 to 100 Hz in α_2_δ3^−/−^ IC units. The loss of precision of temporal coding with increasing f_m_ from 70 to 160 Hz was characterized using a normalized offset-corrected (Pearson-like) correlation coefficient, which appeared more appropriate than the metrics of vector strength. The processing deficits of AM sounds analyzed at the level of the IC indicate that α_2_δ3^−/−^ mice exhibit a subcortical auditory processing disorder (APD). Similar deficits may be present in other mouse models for ASDs.

## Significance Statement

Human speech and animal vocalization contain amplitude-modulated (AM) sounds. Perception of AM sounds requires correct processing of auditory signals in cochlea, auditory brainstem and the inferior colliculus (IC). We have analyzed the ability of IC neurons (units) to extract AM sounds in mice lacking the *Cacna2d3* gene encoding the α_2_δ3 subunit of voltage-activated calcium channels, which are important for proper function of excitatory synapses. With increasing modulation frequencies, units of α_2_δ3^−/−^ mice showed reduced evoked activity, less precise temporal coding, and longer response latencies compared with units of α_2_δ3^+/+^ mice. Humans lacking *Cacna2d3* exhibit genetic autism, a neurodevelopmental disorder often accompanied by impaired auditory processing. We propose *Cacna2d3* as a candidate gene linking an auditory processing disorder (APD) with genetic autism.

## Introduction

Mammals have evolved an extremely powerful auditory system as their communication largely relies on producing and decoding signals from conspecifics, i.e., vocalizations in animals or speech in humans (Woolley and Portfors, 2013; Knörnschild, 2014). Sensory information transduced by hair cells and transmitted from cochlear spiral ganglion neurons in the cochlea is processed by multiple auditory nuclei of the brainstem (cochlear nuclear complex, superior olivary complex, nuclei of the lateral lemniscus), the midbrain (inferior colliculus; IC), and the auditory thalamus before the information enters the cortex for perception (Malmierca and Merchán, 2004). Specialized networks of the auditory brainstem extract features of acoustic information, e.g., onset, offset, space, pitch, and periodicities such as amplitude modulations (AMs) and carrier fine structure (Eggermont, 2015). Notably, vocalization/speech sounds are comprised of AM signals. The aim of the study was to analyze synchronized neuronal responses (phase-locking) evoked by AM tones. These are typically restricted to about f_m_ = 200 Hz as shown by others for the rat (100–200 Hz), the guinea pig (below 150 Hz; for review, see Joris et al., 2004), and for mice (below 200 Hz; Walton et al., 2002).

The central part of the IC contains units that are specialized for processing temporally modulated sound (Langner, 1992; Krishna and Semple, 2000). Recently it became clear that processing of complex sounds is largely performed by the auditory brainstem and the IC, i.e., in subcortical regions (Pressnitzer et al., 2008; Pannese et al., 2015; Felix et al., 2018; Kopp-Scheinpflug and Linden, 2020).

(Central) auditory processing disorders ([C]APD) are characterized by an impaired capacity to discriminate complex or rapidly changing sounds such as speech despite normal hearing thresholds (American Academy of Audiology Clinical Practice Guidelines, 2010; Bellis and Bellis, 2015; Wilson, 2019; Dillon and Cameron, 2021). Genetic forms of APD studied in mice have revealed that even small changes in first spike latency (FSL) or their variability (jitter) in auditory processing can lead to APD. Candidate genes code for cytoskeletal components (beta4spectrin), synaptic proteins (complexin), or ion channels such as K_v_1.1, Kv3.3, α7-nAChR, and α_2_δ3 (Kopp-Scheinpflug and Tempel, 2015; Felix et al., 2019).

The gene *Cacna2d3* encodes the α_2_δ3 auxiliary subunit of voltage-gated Ca^2+^ channels (Catterall et al., 2005; Dolphin, 2012). α_2_δ3 mRNA is strongly expressed in spiral ganglion neurons, in the dorsal and ventral cochlear nucleus, the superior olivary complex, and in some neurons of the IC (Cole et al., 2005; Neely et al., 2010; Pirone et al., 2014). Mice lacking *Cacna2d3* show nearly normal hearing thresholds and normal hair cells yet distorted waveforms of auditory brainstem responses (Neely et al., 2010; Pirone et al., 2014). Notably, malformed and functionally impaired auditory nerve (endbulb of Held) synapses resulted in decreased growth functions and increased FSL of 1.0 ms of postsynaptic action potentials (APs) of bushy cells in the ventral cochlear nucleus in these mice (Pirone et al., 2014). In an auditory discrimination learning experiment, α_2_δ3^−/−^ mice were able to discriminate pure tones (PTs; 7 vs 12 kHz) but failed to discriminate AM tones with a carrier of 12 kHz and modulation frequencies (f_m_) of 20 versus 40 Hz (Pirone et al., 2014).

Loss of function mutations of *Cacna2d3* have been recently identified as risk mutations for autism spectrum disorders (ASDs) in humans (Iossifov et al., 2012; Girirajan et al., 2013; De Rubeis et al., 2014). Notably, children with ASD frequently show impaired sensory processing, especially impaired auditory processing at the level of the auditory brainstem and midbrain (Alcántara et al., 2012; O’Connor, 2012; Robertson and Baron-Cohen, 2017; Scott et al., 2018; Jones et al., 2020).

In this study we examined neuronal responses as single- and multi-units recorded in the IC, which are highly responsive to AM tones, in ketamine-xylaxine anesthetized mice. Whereas spectral processing was not affected in *Cacna2d3*-deficient mice, temporal processing was impaired resulting in a reduced ability to follow AM tones with f_m_ > 70 Hz. Overall, mice lacking the gene *Cacna2d3*, a risk gene for ASD, represent a model for a subcortical APD.

## Materials and Methods

### Animals

Mice with a targeted deletion of the *Cacna2d3* gene coding for α_2_δ3 with insertion of a bacterial β-galactosidase under its promoter (B6.129P2-Cacna2d3tm1Dgen) were generated by Deltagen (Neely et al., 2010) and purchased through The Jackson Laboratory. They were crossed on a C57Bl/6N background (Charles River) for at least 10 generations. For electrophysiological recordings, seven α_2_δ3^−/−^ mice (knock-out, five males, two females) and five α_2_δ3^+/+^ (wild type, two males, three females) of both sexes from heterozygous breedings (littermates) at the age of 12 ± 2 weeks were used. Animals were housed in a temperature-controlled animal facility with free access to food and water and a 12/12 h light/dark cycle.

The animal care, use and experimental protocols followed the national and institutional guidelines, and were reviewed and approved by the Animal Welfare Commissioner and the Regional Board of Animal Experimentation. All experiments were performed in accordance with the European Communities Council Directive (86/609/EEC).

### Surgical procedure

A ketamine-xylazine anesthesia was injected intraperitoneally before performing the surgery and recording. The initial mixture was 6 mg/kg xylazine (Rompun 2%; Bayer Vital), 120 mg/kg ketamine (Ketamin 10%; Bela-Pharm GmbH & Co KG), and 0.16 mg/kg atropine sulfate (B. Braun Melsungen AG). During surgery and recordings, the adequate anesthetic level was maintained by injecting 30% of the initial anesthesia mixture about every 20 min. Body temperature was maintained at 38°C with a custom-made feedback-controlled heating pad. Fur, skin and periosteum were removed from the dorsal surface of the skull. A bonding agent (Gluma Comfort Bond; Heraeus Kulzer) was spread over the fixed skull and a 3-cm-long aluminum bar weighing 0.4 g (for head fixation during the experiment) was fixed on the frontal bones with UV-hardening dental cement (Ivoclar vivadent). An insect needle (diameter, 0.25 mm; Fine Science Tools) serving as reference electrode was inserted into the skull touching the brain.

### Acoustic stimulation and stimulus program

Recordings of the animals were performed in an anechoic, sound-attenuated chamber. The experimental sessions lasted between 8 and 22 h. The animals were killed at the end of the experiment by an overdose of the narcotic mixture.

Acoustic stimuli were presented free-field via a loudspeaker (Schallwandler W06; Manger). The distance from the animal’s head to the loudspeaker was 30 cm with an angle of 45°. To measure the speaker’s output a condensor microphone (Brüel & Kjær 4135; Brüel & Kjær) was placed between the ears. The signal of the microphone was monitored with a measuring amplifier (Brüel & Kjær 2633; Brüel & Kjær) and read in dB sound pressure level (SPL). The frequency spectrum was controlled with a spectrum analyzer (Ono Sokki Multi-purpose FFT Analyzer CF-5220; Ono Sokki Technology). In the intensity range from the microphone’s noise floor (23.5 dB SPL) to the highest amplitude of the presented tones (70 dB SPL), no additional distortions were detected. Acoustic stimuli (PTs and AM tones) were generated with an NI-PCI 6711 card (National Instruments) and controlled with MATLAB software (MATLAB version 7.3.0 R2006b; The MathWorks). After the signal was transmitted via a BNC unit (BNC-2120; National Instruments) to a computer-controlled attenuator (gPAH; g-tec) it was sent via an audio amplifier (Denon, PMA-1060) to the loudspeaker. The frequency characteristics of the loudspeaker was adjusted by ±5 dB by the software in the whole frequency range used in the experiments (1–64 kHz).

For the acoustic stimulation, PTs were presented for 200 ms (including 5-ms rise and fall times) randomly with 15 repetitions for each frequency at constant intensity of 70 dB SPL and an intertone interval of 1000 ms. The frequency range was split into 16 logarithmically spaced frequencies to determine the best frequency (BF) of the unit. The individual BF of every unit is the frequency with the highest evoked rate, and was taken as carrier frequency (f_c_) for the stimulation with AM tones. For AM tones, the same adjustments for SPL (70 dB, which was well above the hearing thresholds respectively the thresholds of the CF) and intertone interval were applied but the stimulus duration was 500 ms. The modulation frequency (f_m_) intervals of AM tones were adapted by dividing them into 20 parts in steps of 5 or 10 Hz depending on the phase-locking of the unit (see below). All AM tones were sinusoidally modulated signals with a modulation depth of 100%.

Tuning curves, which describe the sensitivity of a unit as a function of frequency, were recorded using a tone duration of 100 ms. Each tone was presented randomly at 16 logarithmically spaced frequencies in the frequency range of 1–64 kHz with 10 repetitions each with an interstimulus interval of 600 ms. SPL ranged from 0 to 70 dB SPL in steps of 10 dB to determine the unit’s receptive field.

### Electrophysiological recordings

Recordings were performed from both sides of the IC in a stereotactic frame. Recording depth ranged from 200 to 1600 μm from the surface. In total, we recorded from 162 single- and multi-units of α_2_δ3^−/−^ and from 179 single- and multi-units of α_2_δ3^+/+^ mice. Tungsten electrodes (impedance 1 MΩ; Microelectrode Tungsten Kapton, TM 33A10KT; World Precision Instruments) were inserted orthogonally into the IC with a micromanipulator (MM 33; Märzhäuser). For each further recording site, the electrodes were advanced in depth in 200-μm intervals. Extracellular neuronal signals were collected and amplified via a headstage (HST/8o50-G1-GR Omnetics, Headstage; Plexon), transmitted to a preamplifier and bandpass filter (PBX2/16SP-G50; Plexon; 50 000-fold amplification; filter bandwidth, 100 Hz to 8 kHz), and sent to a recording system (MAP; Plexon) and oscilloscope (Yokogawa DL 708E) for audiovisual control of the recordings.

To characterize the units by their response patterns to the acoustic stimulation, a spike analysis software (sort client version 2.3.4; Plexon) was used. Offline-sorting with principal component analysis accepted single- and multi-units up to three units. After stimulation with PTs, the BF of each unit was estimated through the highest evoked rate, which was determined after subtracting the spontaneous activity. Thereafter, the units were stimulated with AM tones up to an f_m_ of 100 or 200 Hz, respectively. To identify the highest modulation frequency at which the neuronal responses were still synchronous (phase-locked) with the AM stimulation, the vector strength (Goldberg and Brown, 1969; Greenwood, 1986) was calculated for a first quick evaluation. The distribution of the neuronal responses was checked for randomness using a Rayleigh test for every modulation frequency at a significance level of 0.01 (Batschelet, 1965; Mardia, 1972). Those units that phase-locked up to f_m_ = 100 Hz were further stimulated up to 200 Hz in 10-Hz steps.

### Data analysis

Reponses to stimulation with PTs and AM tones were recorded and analyzed with MATLAB (for PTs) and custom software implemented in C++ (for AM tones). For PT stimulation, the BF was determined, which is the tone frequency that generates the highest evoked discharge rate.

For AM tones, we calculated several parameters describing temporal characteristics of all units and of the population, respectively. For every f_m_ from 10 to 160 Hz in steps of 10 Hz, we searched for those units in which the f_c_ was equal to the BF in both genotypes. First, we calculated the spontaneous discharge rate of the population, i.e., the discharge rate of the population without a stimulus, as mean value across 15 repetitions of all units in a period of 500 ms before the AM stimulus. The spike times of every unit and for the population were binned into a peristimulus time histogram (PSTH) with 200-μs resolution. All further calculations were done with this PSTH. Then we calculated the FSL of a unit, which is defined by the time when the first spike of a unit elicited by the stimulus exceeded its spontaneous rate by 2 SDs. The median of all unit’s FSL is the FSL of the population (Anderson and Linden, 2016). To calculate various results of the neuronal responses to a stimulus both per period of the AM (evoked rate, peak latency) and during the entire stimulus (peak latencies, vector strength, correlation coefficients), spike trains of individual units and population were convolved by a Gaussian kernel σ (Eq. 1):

(1) σ=80 Hzfmms,

superposed and then compiled to a convolved population PSTH (CPSTH) with time bins of 200 μs. This Gaussian kernel is similar as the one used by Bendor and Wang (2007), however, our SD is a function of the f_m_ to avoid too strong smoothing with increasing f_m_ on the one hand and keep a reasonable resolution on the other hand. Minimum points of this CPSTH define start and end of the periods in response to the stimulus. After that, the evoked rate of the population per period was calculated across all periods of AM stimuli by subtracting spontaneous discharge rate plus 2 SDs. Further, we calculated the vector strength of the population without those periods of the response corresponding to the first 100 ms to avoid distortions by the onset response. After peak latencies had been calculated per period (omitting the first 100 ms) for every unit, the peak latency and jitter of the population and the mean peak latency were calculated. Peak latency was defined as the time lag from the period’s start of the stimulus to the dedicated maximum of a normalized cross-correlation histogram (NCCH). NCCHs were calculated with one period of the stimulus as a reference signal (r[*n*]) and the PSTH of (1) every unit and (2) of the population, respectively (see Eq. 2):

(2) $$NCCH\left[k\right]=\frac{\sum_{n=1}^{N}r\left[n\right]\cdot PSTH[n+k]}{\sqrt{\sum_{n=1}^{N}{\left(r[n]\right)}^{2}}\cdot \sqrt{\sum_{n=1}^{N}{\left(PSTH\left[n\right]\right)}^{2}}}.$$

Normalization of the cross-correlation function was performed without those periods corresponding to the first 100 ms of the neuronal response as was done in calculating the vector strength. The peak latency of the population is the mean value of the individual period’s peak latencies of the population, and the jitter is defined by the SD. Mean peak latency was calculated as the mean of the peak latencies of each individual unit. In addition, we calculated peak latencies by the time lag from the period’s start of the stimulus to the dedicated maximum in the CPSTH. Finally, we used the highest correlation coefficient per period of the NCCH as an additional information to assess the neuronal response. For each modulation frequency, we calculated a mean cross-correlation coefficient CC_NC_ as mean value of the coefficients per period. The reference signal as well as the neuronal response have only positive values, so the calculated mean cross-correlation coefficients are in a possible range of 0 (no correlation) and 1 (identical signal). As the modulation frequency increases, the periodic time of the modulation signal respectively the reference signal decreases. The ratio of the duration of the reference signal to the duration of the examined periods (400 ms) of the neuronal response therefore becomes smaller and smaller and so do the correlation coefficients. To compensate for this effect, mean correlation coefficients at different modulation frequencies were scaled by multiplying them with the square root of the number of the considered periods (see Eq. 3):

(3) $$CC_{}=(\frac{1}{M}\cdot \overset{M}{\underset{i=1}{\sum }}CC\left[i\right])\cdot \sqrt{M}.$$

A cross-correlation would result in positive values even if the waveform per period was completely flat with evoked rates >0. Therefore, we performed the same calculations with offset-corrected signals to avoid this effect. As a result, the reference signal became symmetrical to the zero baseline (s[*n*]). Further, the mean of the considered periods of the PSTH (400 ms) for every modulation frequency was subtracted from those PSTHs. The principle of calculation is basically identical to the calculation of Pearson’s correlation coefficient, resulting in a Pearson-like correlation histogram (PCH) with values ranging from −1 to 1 (Eq. 4):

(4) $$PCH\left[k\right]=\frac{\sum_{n=1}^{N}s\left[n\right]\cdot (PSTH\left[n+k\right]-\bar{PSTH})}{\sqrt{\sum_{n=1}^{N}{\left(s[n]\right)}^{2}}\cdot \sqrt{\sum_{n=1}^{N}{\left(PSTH\left[n\right]-\bar{PSTH}\right)}^{2}}}.$$

The Pearson-like (mean) correlation coefficients (CC_PC_) was calculated according to Equation 3.

To analyze tuning curves the following parameters were determined: (1) characteristic frequency (CF), frequency at which the sound level that evoked a response significantly larger (3 SDs) than spontaneous firing rate was minimal; (2) threshold in dB SPL at CF; and (3) calculation of Q40 value, frequency bandwidth of the tuning curve 40 dB SPL above the CF. The Q40 value was calculated as the ratio between the CF of the tuning curve and the bandwidth of the tuning curve 40 dB above the CF.

### Statistical analysis

Statistical analyses were performed with Statistica 13.3 (StatSoft). All data were tested for normal distribution. As most of the data were not normally distributed, they were analyzed using the Mann–Whitney *U* test. All tests were two-tailed with α = 0.05.

## Results

This study is based on analyses of single- and multi-unit recordings in the IC from both sides of five α_2_δ3^+/+^ and seven α_2_δ3^−/−^ mice. The percentage of single-units of all recorded units was very similar in both genotypes (25% in α_2_δ3^+/+^ vs 21% in α_2_δ3^−/−^). In total, we evaluated 179 α_2_δ3^+/+^ and 162 α_2_δ3^−/−^ single- and multi-units each including PT stimulation with SPLs from 0 to 70 dB SPL in steps of 10 dB and experiments using AM tone stimulation with different carrier and modulation frequencies at 70 dB SPL. For AM tone stimulation, 97 α_2_δ3^+/+^ units and 99 α_2_δ3^−/−^ units were analyzed up to modulation frequencies (f_m_) of 100 Hz, and 77 α_2_δ3^+/+^ units respectively 73 α_2_δ3^−/−^ units for modulation frequencies above. Only those units of both α_2_δ3^+/+^ and α_2_δ3^−/−^ animals were selected in which the f_c_ was identical to the BF of either 2828, 4757, 5657, 6727, 8000, 9514, 11,310, 13,450, 16,000, or 19,030 Hz. All units responded to PT stimulation and had BFs between 2 and 45 kHz. The mean BF was 11.6 kHz for α_2_δ3^+/+^ and 11.8 kHz for α_2_δ3^−/−^. There was no statistically significant bias in BF for any distribution of response parameters (tone response types, evoked response, Mann–Whitney *U* test).

In our experimental design, we did not measure hearing thresholds of the mice, e.g., by auditory brainstem responses before IC recordings. However, we determined thresholds from tuning curves at the CF of all units, which revealed thresholds elevated by 5 dB ± 5 dB in α_2_δ3^−/−^ compared with α_2_δ3^+/+^ units. Further, at the frequency of best hearing in mice, 11,310 Hz, the median threshold of α_2_δ3^+/+^ units amounted to 20 dB SPL whereas those of α_2_δ3^−/−^ units were 30 dB SPL (*p *=* *0.048, Mann–Whitney *U* test). Although α_2_δ3^−/−^ units showed slightly higher thresholds, their frequency range (CFs registered from 4757 Hz to 26,910 Hz, *n* = 91) was not corrupted compared with α_2_δ3^+/+^ (from 4757 Hz to 19,030 Hz, *n* = 81). The median of all CFs was 11,310 Hz for both α_2_δ3^+/+^ and α_2_δ3^−/−^, with 25th percentiles of 9514 Hz for both α_2_δ3^+/+^ and α_2_δ3^−/−^, and with 75th percentiles of 13,450 Hz (α_2_δ3^+/+^) and 16,000 Hz (α_2_δ3^−/−^).

### Spontaneous rate and spectral properties of the neuronal responses

**Figure 1.**
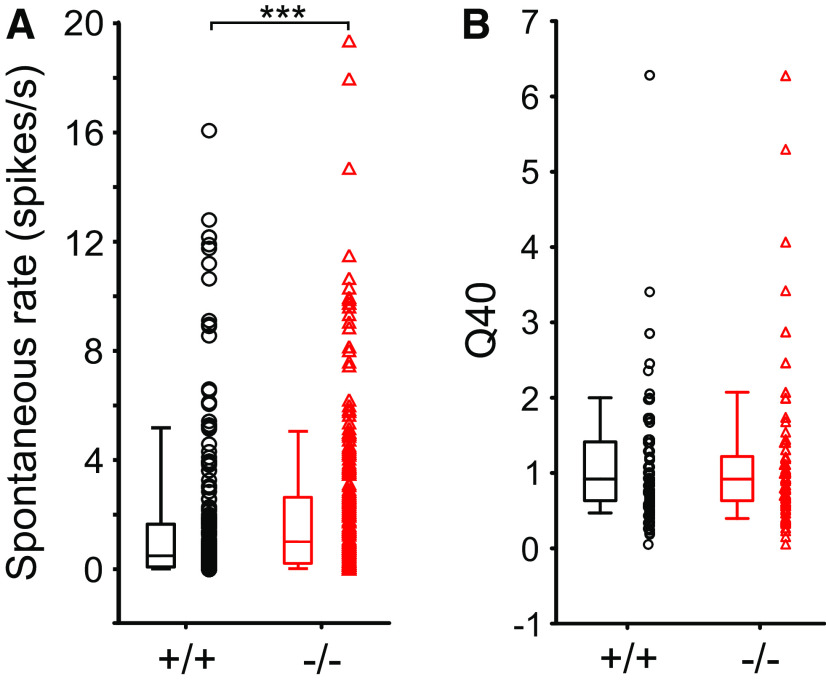
Increased spontaneous discharge rate yet unaltered sharpness of tuning in α_2_δ3^−/−^ mice. ***A***, Spontaneous discharge rates of units from α_2_δ3^+/+^ (+/+, black) and α_2_δ3^−/−^ mice (−/−, red) obtained from extracellular recordings in the IC. Individual data and box plots with medians (horizontal lines), interquartile ranges (25% and 75%, boxes), and whiskers (10%, 90%) of 223 α_2_δ3^+/+^ and 261 α_2_δ3^−/−^ units revealed an increased spontaneous rate (*p *=* *0.0007, Mann–Whitney *U* test, *** *p* < 0.001). ***B***, There was no difference in the sharpness of frequency tuning expressed as Q_40dB_ values between 154 α_2_δ3^+/+^ and 119 α_2_δ3^−/−^ units (*p *=* *0.79, Mann–Whitney *U* test).

The spontaneous discharge rate of the units (SR), which is shown by individual data and box and whisker plots, varied from 0 to 19.4 spikes/s with a median of 0.49 spikes/s for α_2_δ3^+/+^ and 1.02 spikes/s for α_2_δ3^−/−^ mice (Fig. 1*A*). Thus, the SR was 2.08-fold higher in α_2_δ3^−/−^ compared with α_2_δ3^+/+^ mice (*p *=* *0.0007, Mann–Whitney *U* test). Similar results were determined from prestimulus periods in the AM-tone experiments (data not shown).

The Q40 value is a dimensionless measure of tuning sharpness, with higher values indicating that a filter is more sharply tuned (Capranica, 1992). In general, Q40 values were rather small (broad tuning) but they did not significantly differ between α_2_δ3^−/−^ compared with α_2_δ3^+/+^ mice as shown in a box and whisker plot (Fig. 1*B*). Median Q40 values for both α_2_δ3^+/+^ and α_2_δ3^−/−^ were 0.92 (*p *=* *0.79, Mann–Whitney *U* test).

### Temporal properties of the neuronal responses

**Figure 2.**
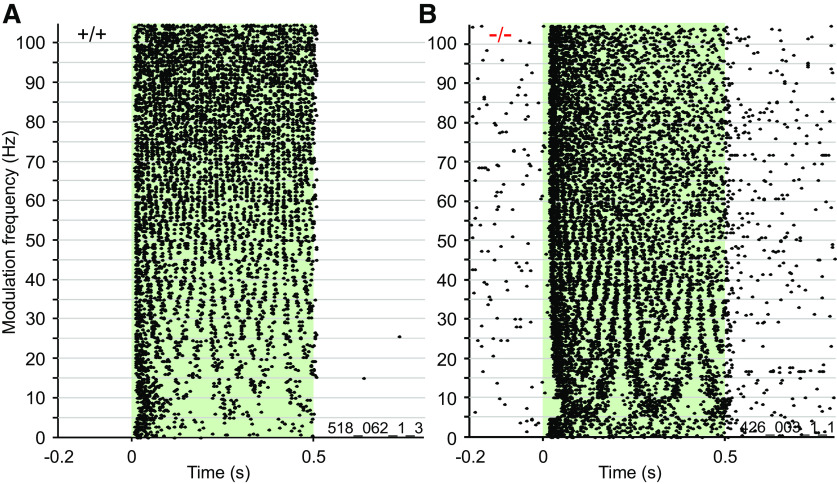
Examples of neuronal responses to AM tone stimulation of α_2_δ3^+/+^ and α_2_δ3^−/−^ mice. ***A***, Raster plot from an α_2_δ3^+/+^ unit responding with action potentials (dots) to AM tone stimulation of modulation frequencies between 0 and 100 Hz showing an onset response and subsequent phase locking over the entire range of AMs (significant vector strength, Rayleigh test, *p* = 0.01). ***B***, Raster plot from an α_2_δ3^−/−^ unit showing a similar response with phase-locking up to 70-Hz modulation frequency (significant vector strength, Rayleigh test, *p* = 0.01). In both examples, the carrier frequency was set to the BF of the unit, here 11,310 Hz. Responses to each modulation frequency are represented by 15 horizontal rows of dots corresponding to 15 trials. The green area indicates the duration of the stimulus.

**Figure 3.**
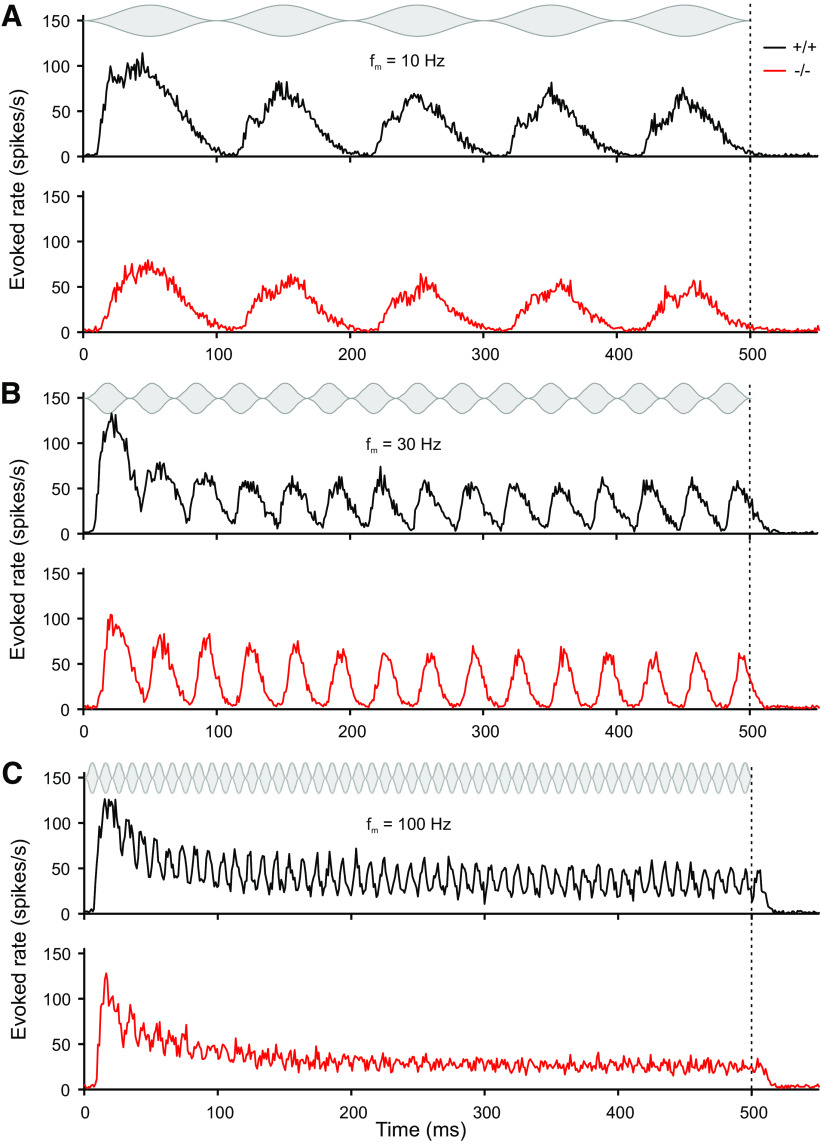
IC units of α_2_δ3^−/−^ mice lose precision of temporal coding and show a reduced evoked rate to higher modulation frequencies. ***A–C***, Evoked rate (activity averaged over all AM tone stimulations at a particular modulation frequency) for all units with carrier frequency of the AM tone equal to their BF of α_2_δ3^+/+^ (+/+, black, *n* = 97) and α_2_δ3^−/−^ (−/−, red, *n* = 99) mice as a function of time. Averaged evoked rate (PSTH) is shown for f_m_ = 10 Hz (***A***), f_m_ = 30 Hz (***B***), and f_m_ = 100 Hz (***C***). The AM stimulus with 500-ms duration is shown above each panel. This figure was created using a binning time of 1 ms.

Units in the IC typically show discharges in response to AM tones that are phase-locked to the f_m_ of the stimulus. Therefore, the temporal structure of the sound is encoded in the temporal structure of the neuronal discharge rate. Figure 2 shows examples of an α_2_δ3^+/+^ (Fig. 2*A*) and an α_2_δ3^−/−^ unit (Fig. 2*B*) each with typical phase-locked responses (raster plot) to the envelope of the AM tone stimulus for f_m_ between 0 Hz (unmodulated carrier) and 100 Hz. In both examples, the f_c_ was set to the BF of the unit, here 11,310 Hz. Each horizontal row of dots represents the responses to one f_m_ with 15 trials each; the green area indicates the duration of the stimulus. The α_2_δ3^+/+^ unit (Fig. 2*A*) displays significant phase locking throughout the range of modulation frequencies tested (vector strength, Rayleigh test, *p* = 0.01) over the entire range of AM. An α_2_δ3^−/−^ unit (Fig. 2*B*) responded in a similar way with phase locking up to a f_m_ of 70 Hz (vector strength, Rayleigh test, *p* = 0.01). All neuronal responses to AM tone stimuli were binned into a PSTH. Figure 3 shows examples for PSTHs for f_m_ of 10 Hz (Fig. 3*A*), 30 Hz (Fig. 3*B*), and 100 Hz (Fig. 3*C*) as a function of time. The gray plot above each panel indicates the envelope of the AM tone. Using these PSTHs and the start and end of periods of the responses determined with the corresponding convolved PSTHs (CPSTHs), evoked rates per period were calculated for the population.

**Figure 4.**
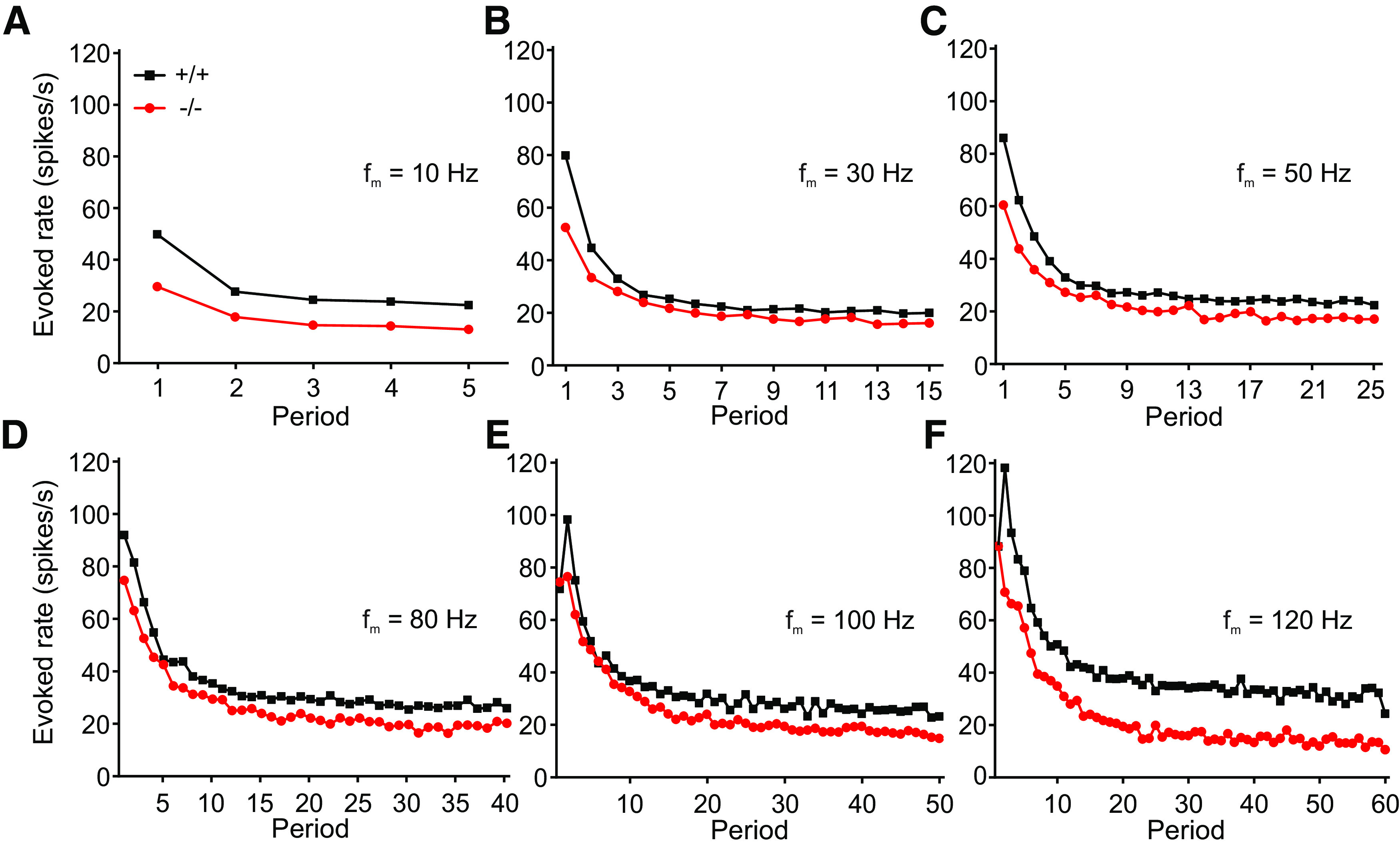
AM tone-evoked rates of the population were lower in α_2_δ3^−/−^ mice compared with α_2_δ3^+/+^; the difference of evoked rates between genotypes increased on high modulation frequencies. ***A–F***, AM tone-evoked rates of IC units from α_2_δ3^+/+^ (+/+, black, *n* = 97 for f_m_ ≤ 100 Hz; *n* = 77 for f_m_ > 100 Hz) and α_2_δ3^−/−^ (−/−, red, *n* = 99 for f_m_ ≤ 100 Hz; *n* = 73 for f_m_ > 100 Hz) mice as a function of the period of the respective f_m_ and for selected modulation frequencies. The onset response is visible in the first periods of each modulation frequency.

To determine whether the evoked rate of the population was sensitive to higher f_m_ and rising period numbers of the stimulus in either genotype, we compared the evoked rate of the population for f_m_ varying from 10 to 160 Hz shown for selected f_m_ in Figure 4*A–F*. There was increasing variation over periods in evoked rates for higher f_m_. The change in the evoked rate of the population for either low (10 Hz) or high (120 Hz) frequency modulations as a function of the period had a similar shape in both genotypes but always revealed lower rates in α_2_δ3^−/−^ units. Note that for each f_m_, an onset response is visible in the first periods reflecting the well-known adaptation phenomenon for longer duration of the stimulus.

**Figure 5.**
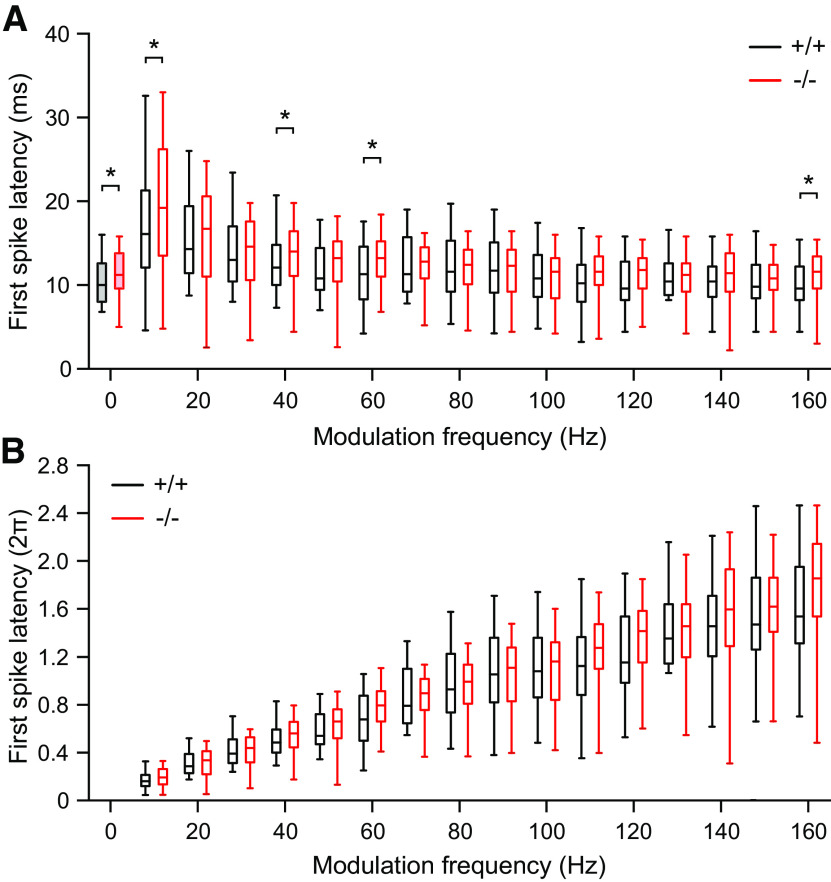
Median FSLs were longer in IC units of α_2_δ3^−/−^ compared with α_2_δ3^+/+^ mice at some modulation frequencies. FSLs (***A***) and FSLs in relation to the periodic time (***B***) of neuronal responses to AM tone stimulation of α_2_δ3^+/+^ units (+/+, black) and α_2_δ3^−/−^ units (−/−, red) as a function of modulation frequency. Box plots with medians (horizontal lines), interquartile ranges (25% and 75%, boxes) and whiskers (10%, 90%) of FSL. FSL of the neuronal response to the unmodulated carrier frequency (0 Hz, PT, left, filled box plot) and to different modulation frequencies (10–160 Hz) for α_2_δ3^+/+^ (*n* = 97 for f_m_ ≤ 100 Hz; *n* = 77 for f_m_ > 100 Hz) and α_2_δ3^−/−^ units (*n* = 99 for f_m_ ≤ 100 Hz; *n* = 73 for f_m_ > 100 Hz; Mann–Whitney *U* test, *p < 0.05).

Next, we calculated FSLs for f_m_ from 10 to 160 Hz and for PT (f_m_ = 0 Hz) as shown by a box-and-whisker plot in ms (Fig. 5*A*) and, for a better representation of the differences in latency, especially at higher f_m_, in multiples of the periodic time (Fig. 5*B*). Median FSLs in response to different f_m_ were always longer in α_2_δ3^−/−^ compared with α_2_δ3^+/+^ mice. However, there were only few significant differences such as for the f_m_ of 10 Hz (*p *=* *0.016), 40 Hz (*p *=* *0.031), 60 Hz (*p *=* *0.002), 160 Hz (*p *=* *0.012) as well as 0 Hz (Mann–Whitney *U* test; Fig. 5*A*), which is likely caused by the large variations in FSL. FSL values of the unmodulated tone (PT, f_m_ = 0 Hz; filled gray and red box) were shorter in both genotypes compared with the respective FSL values in all cases of f_m_ because of the very short stimulus ramp of the PT lasting 5 ms. For the PT stimulation, median FSL was significantly larger in α_2_δ3^−/−^ mice (*p *=* *0.036; Fig. 5*A*).

Peak latencies and jitter were calculated as the time lag from the start of the stimulus within a period to the dedicated maximum in the normalized cross-correlation function and in the CPSTH (see Materials and Methods, Data analysis), respectively. Here, we show the results of the cross-correlation functions only because results from the maxima of the CPSTH were very similar.

**Figure 6.**
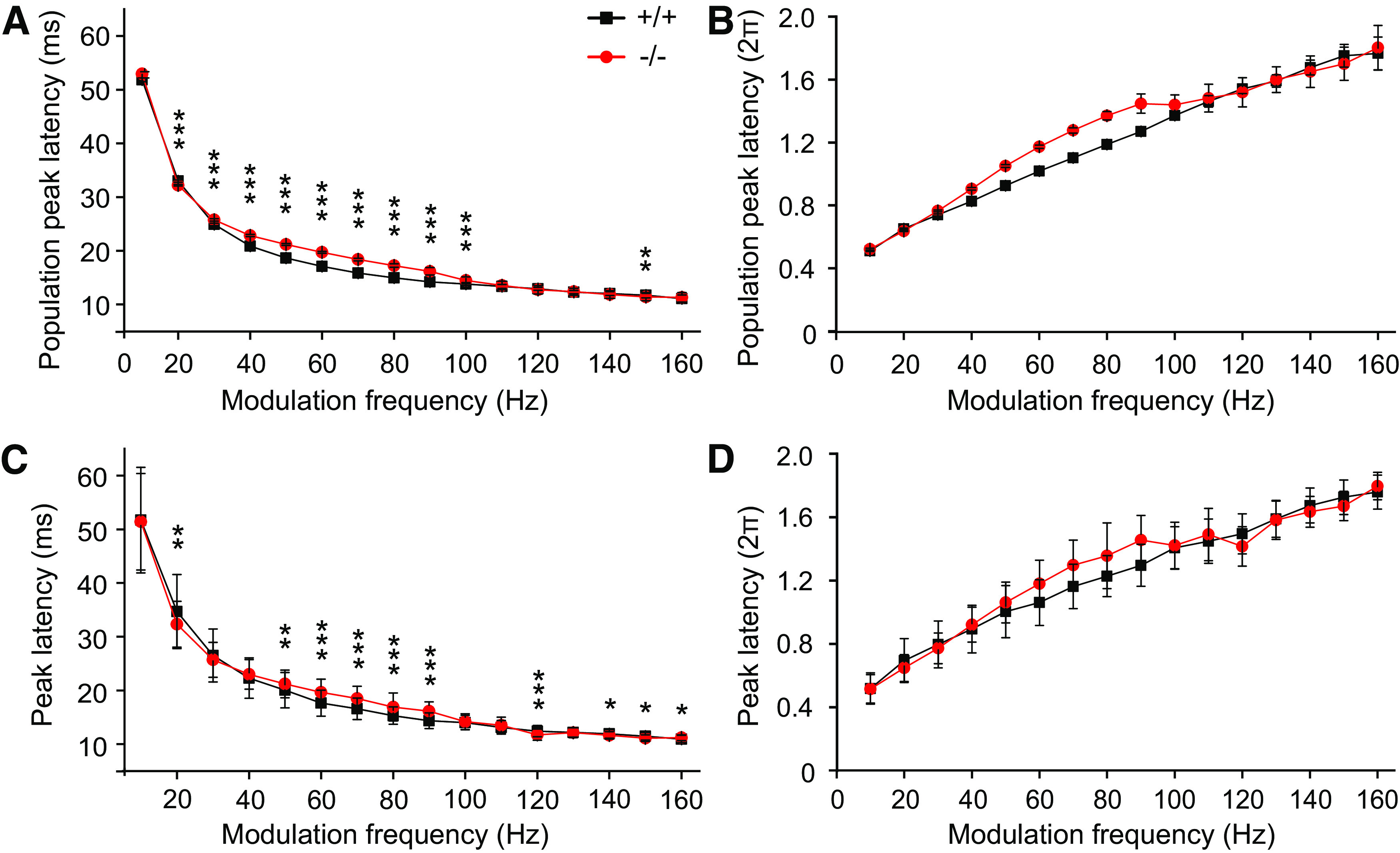
Peak latencies of IC units from α_2_δ3^−/−^ mice are longer compared with those from α_2_δ3^+/+^ mice. Population peak latency ± SD (jitter; ***A***) and population peak latency ± SD (jitter; ***B***) in relation to the periodic time of neuronal responses to AM tone stimulation for units of α_2_δ3^+/+^ (+/+, black) and α_2_δ3^−/−^ mice (−/−, red). ***C***, Mean peak latency ± SD calculated from each of neuronal responses to AM tone stimulation and each period. ***D***, Mean peak latency ± SD in relation to periodic time of AM tone stimulation. *p < 0.05; **p < 0.01; ***p < 0.001.

Figure 6*A*,*B* and Table 1 show the peak latency of the population as a function of f_m_ for both genotypes each with SD (jitter). Whereas Figure 6*A* shows the absolute values of the peak latency in milliseconds, it was divided by the periodic time of neuronal responses to AM tone stimulation in Figure 6*B*. The advantage of this representation is the elimination of the shorter absolute latency with increasing f_m_. Peak latencies were significantly larger in α_2_δ3^−/−^ units for f_m_ from 20 Hz to 100 Hz (*p *<* *0.001; Fig. 6*A*). Notably, there was an increasing jitter (SD) with increasing f_m_ visible in Figure 6*B* and Table 1. At f_m_ ≥ 100 Hz, we note an approximation of the curves from α_2_δ3^+/+^ and α_2_δ3^−/−^, which is likely caused by the increasingly temporally uncoordinated response to the stimulus in α_2_δ3^−/−^ mice (compare Fig. 3*C*). Mean peak latencies ± SD are shown in Figure 6*C*,*D*, where the SD is a measure for the variations in peak latency within the units. Peak latencies of the population and mean peak latencies have a similar trend. In summary, peak latencies of α_2_δ3^−/−^ were consistently longer compared with α_2_δ3^+/+^ in a large range of f_m_.

**Table 1:** Population peak latencies of IC units from α_2_δ3^+/+^ and α_2_δ3^−/−^ mice as a function of modulation frequency in absolute values and in multiples of the periodic time (Fig. 6*A,B*)

	Peak latency ± SD (ms)		Peak latency ± SD/(2π)	
	α_2_δ3^+/+^	α_2_δ3^–/–^	α_2_δ3^+/+^	α_2_δ3^–/–^
f_m_ (Hz)	Mean ± SD	Mean ± SD	Mean ± SD	Mean ± SD
10	51.75 ± 0.34	52.85 ± 0.57	0.5175 ± 0.0034	0.5285 ± 0.0057
20	32.95 ± 0.21	32.18 ± 0.13	0.6590 ± 0.0041	0.6435 ± 0.0026
30	24.90 ± 0.22	25.75 ± 0.22	0.7470 ± 0.0067	0.7725 ± 0.0066
40	20.85 ± 0.36	22.83 ± 0.20	0.8340 ± 0.0145	0.9130 ± 0.0082
50	18.66 ± 0.24	21.18 ± 0.18	0.9330 ± 0.0122	1.0590 ± 0.0091
60	17.12 ± 0.21	19.69 ± 0.18	1.0270 ± 0.0127	1.1815 ± 0.0105
70	15.88 ± 0.18	18.37 ± 0.25	1.1115 ± 0.0126	1.2860 ± 0.0175
80	14.96 ± 0.26	17.24 ± 0.33	1.1965 ± 0.0208	1.3795 ± 0.0264
90	14.21 ± 0.27	16.19 ± 0.68	1.2790 ± 0.0240	1.4570 ± 0.0615
100	13.81 ± 0.27	14.49 ± 0.64	1.3805 ± 0.0266	1.4490 ± 0.0640
110	13.37 ± 0.34	13.56 ± 0.80	1.4710 ± 0.0373	1.4920 ± 0.0884
120	12.93 ± 0.25	12.74 ± 0.78	1.5515 ± 0.0300	1.5285 ± 0.0933
130	12.32 ± 0.30	12.38 ± 0.63	1.6010 ± 0.0396	1.6095 ± 0.0825
140	12.05 ± 0.34	11.86 ± 0.72	1.6868 ± 0.0476	1.6602 ± 0.1005
150	11.75 ± 0.48	11.41 ± 0.70	1.7622 ± 0.0727	1.7109 ± 0.1056
160	11.11 ± 0.66	11.34 ± 0.89	1.7769 ± 0.1049	1.8137 ± 0.1425

**Figure 7.**
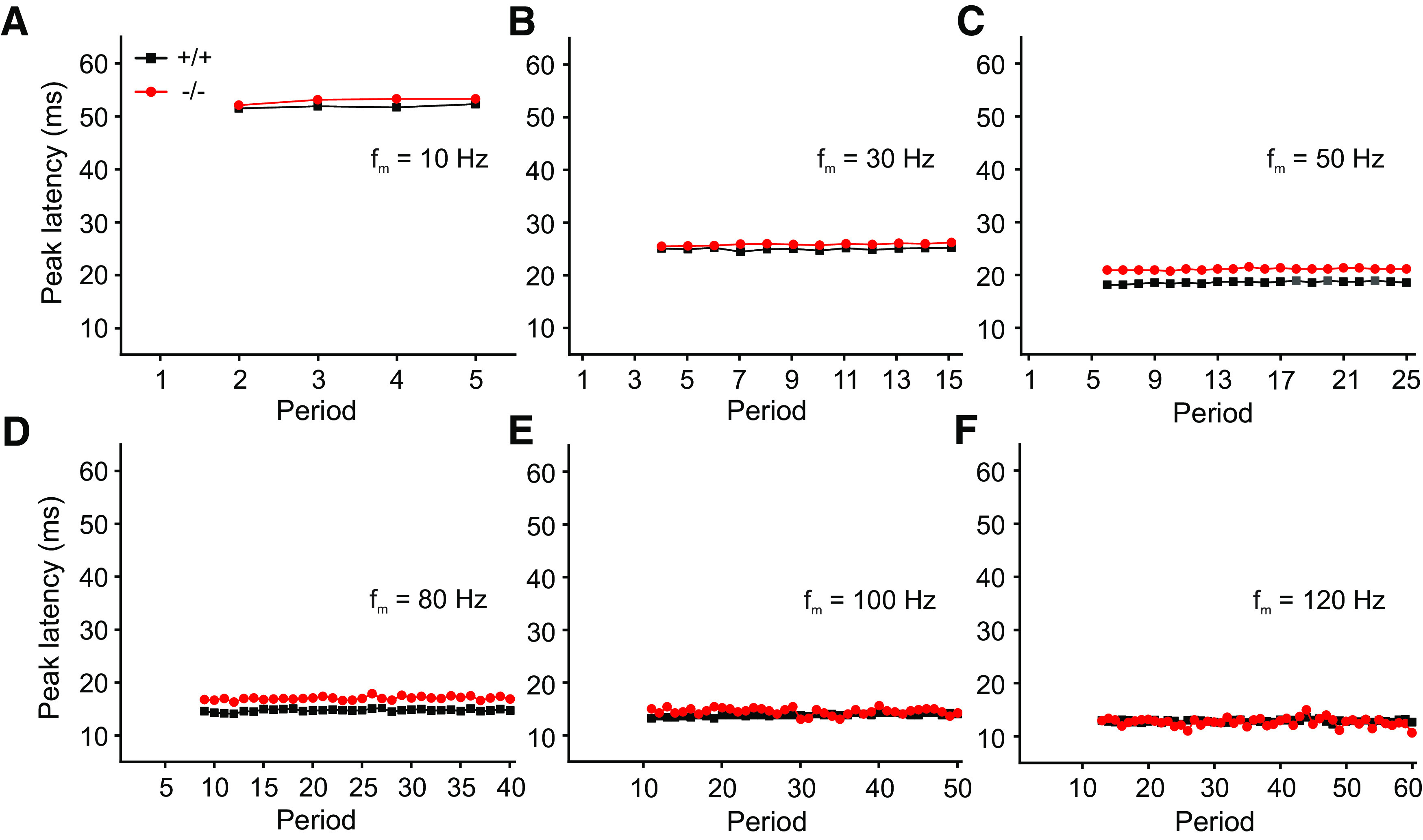
Peak latencies of the population of IC units remain largely constant as a function of period in both α_2_δ3^+/+^ and α_2_δ3^−/−^ mice. ***A–F***, Peak latencies of the population of α_2_δ3^+/+^ (+/+, black) and α_2_δ3^−/−^ units (−/−, red) for selected modulation frequencies are constant over the period of AM tone stimulation for both genotypes except some jitter in panels ***D–F***.

To define how a population of units can follow the stimulus signal over time, we plotted the peak latency of the population as a function of the period for the respective f_m_ for both α_2_δ3^+/+^ and α_2_δ3^−/−^ mice (Fig. 7*A–F*). Apart from small variations, i.e., jitter, which was mainly visible at higher f_m_ – peak latencies of the population were largely constant over the periods for any given f_m_ in both genotypes. Notably, the differences in peak latency between α_2_δ3^+/+^ and α_2_δ3^−/−^ vanish with increasing f_m_. This indicates that there is no loss of temporal coding for peak latencies over periods for α_2_δ3^+/+^ and α_2_δ3^−/−^.

**Figure 8.**
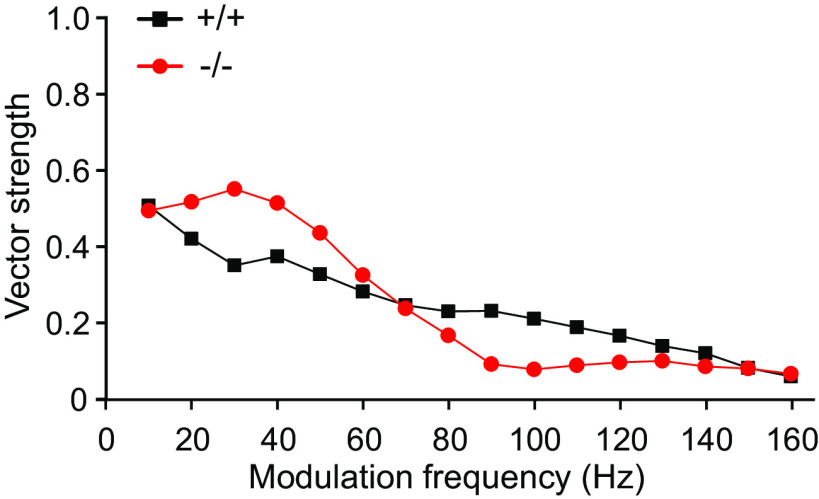
Unexpected dependence of the vector strength of the population on the modulation frequency for IC units from α_2_δ3^−/−^ mice. For modulation frequencies below ∼60 Hz, population vector strength was higher for α_2_δ3^−/−^ units (−/−, red) compared with α_2_δ3^+/+^ units (+/+, black). For f_m_ ∼80 Hz and above, population vector strength of α_2_δ3^−/−^ units declined below that of α_2_δ3^+/+^ units.

Vector strength of the population was calculated using the measured absolute spike times of the units within the determined time window of responses to the 500-ms AM tone stimulation (without the first 100 ms to avoid distortions by onset response). Figure 8 shows the vector strength of population responses to AM tone stimulation as a function of f_m_ from 10 to 160 Hz for α_2_δ3^+/+^ and α_2_δ3^−/−^ mice. At modulation frequencies of ∼80 Hz and above, the vector strength of α_2_δ3^−/−^ was smaller than that of α_2_δ3^+/+^ units, and responses of α_2_δ3^−/−^ units became more and more temporally uncoordinated (see also Fig. 3*C*). Surprisingly, below f_m_ of ∼60 Hz the vector strength of α_2_δ3^−/−^ was larger compared with α_2_δ3^+/+^ (Fig. 8), which apparently indicates a response with better temporal precision (see also Fig. 3*B*). However, this effect does not reflect a more precise response of α_2_δ3^−/−^ units. Rather, in view of the heterogeneity of IC units in general and the prolonged peak latencies of α_2_δ3^−/−^ units (Fig. 6), we suppose that there are phasic units in α_2_δ3^−/−^ mice that cannot respond as quickly as those of α_2_δ3^+/+^ mice at f_m_ of ≳30 Hz (Fig. 3*A*,*B*).

**Figure 9.**
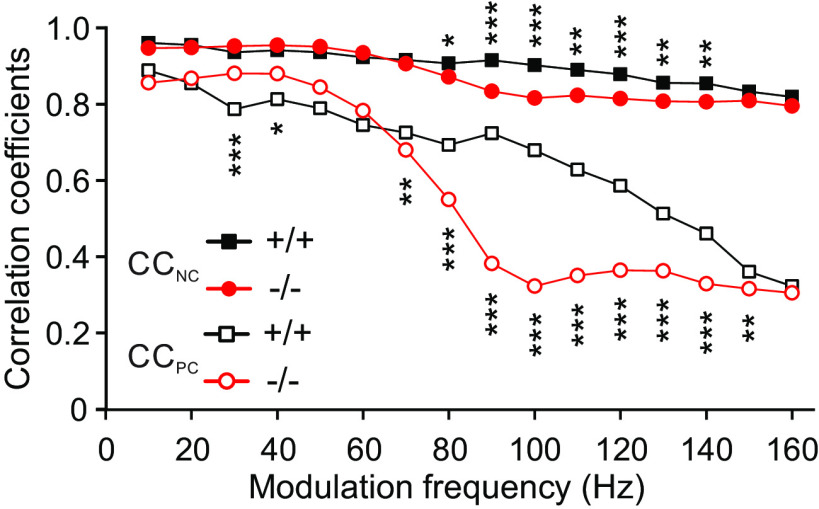
The Pearson-like correlation coefficient is superior to describe the quality of temporal coding. Mean correlation coefficients calculated from NCCH (CC_NC_, closed symbols) were rather similar for α_2_δ3^+/+^ and α_2_δ3^−/−^ IC units because of their dependence on the offset (evoked rate) described under Materials and Methods. For f_m_ ≥ 80 Hz, however, mean CC_NC_ were smaller in α_2_δ3^−/−^ mice as indicated by the stars at the top. In contrast to CC_NC_, mean Pearson-like correlation coefficients (CC_PC_, open symbols) showed a strong decrease for modulation frequencies of ∼70 Hz and above for α_2_δ3^−/−^ compared with α_2_δ3^+/+^ units indicating their markedly reduced ability to follow the stimuli in a coordinated manner (downward stars; * *p *<* *0.05, ** *p *<* *0.01, *** *p *<* *0.001).

In addition to the vector strength, we calculated correlation coefficients (CC_NC_, CC_PC_; see Materials and Methods, Data analysis) for every f_m_ as a measure for the similarity (linear dependence) of stimulus and neuronal response (Fig. 9). The graphs in Figure 9 are shown without SD. Because with increasing period the correlation coefficients have a decreasing tendency because of decreasing evoked rates, this non-random superposition of the deviations of the correlation coefficients causes too high SDs. Their calculation is therefore not meaningful. The coefficients CC_NC_ are similar for α_2_δ3^+/+^ and α_2_δ3^−/−^ mice with a slightly decreasing tendency toward higher f_m_ and smaller coefficients in α_2_δ3^−/−^ mice at ∼80 Hz onwards (closed symbols). Notably, α_2_δ3^+/+^ and α_2_δ3^−/−^ curves show only small differences because of the effect of the offset described in Materials and Methods, Data analysis, “correlation.” In contrast, the correlation coefficients CC_PC_ of the α_2_δ3^−/−^ units (open red symbols) show a strong decrease for modulation frequencies of ∼70 Hz and above indicating a markedly reduced ability to follow the stimuli in a coordinated way as compared with α_2_δ3^+/+^ units (open black symbols). Notably, the CC_PC_ also shows an increase in α_2_δ3^−/−^ compared with α_2_δ3^+/+^ units at f_m_ ∼30 Hz (Fig. 9); this effect is, however, smaller than for the vector strength (Fig. 8). Taken together, the Pearson-like (offset-corrected) cross-correlation method used yielded a more reliable metric (CC_PC_) for the population than the vector strength.

So far, all calculations for AM tone stimuli shown were done with f_c_ equal to the BF (f_c_ – BF = 0). In addition, all calculations were done with a difference of the f_c_ values of a quarter octave and of a half octave to the BF. We found small variations in the results but all conclusions drawn remain valid.

## Discussion

Our results demonstrate temporal processing deficits in the auditory midbrain of α_2_δ3-deficient mice, specifically in neurons of the IC. This adds to our previous findings on malfunctioning synapses in the cochlear nucleus and the inability of auditory discrimination learning of AM tones despite normal hearing thresholds and normal hair cell function in these mice (Pirone et al., 2014).

Despite increased spontaneous rates in α_2_δ3^−/−^ IC units, their evoked rates were reduced compared with α_2_δ3^+/+^ for most modulation frequencies (Fig. 4). Because the IC is the auditory center that extracts and integrates temporal features of auditory signals from the periphery and auditory brainstem nuclei (Eggermont, 2015), we analyzed the responses of IC units to stimulation with AM tones. We found increased FSLs (Fig. 5) and increased peak latencies (Fig. 6) in α_2_δ3^−/−^ IC units for a large range of f_m_. PSTHs revealed that IC units of α_2_δ3^−/−^ mice were able to follow the envelope of the AM tone for lower modulation frequencies similar as IC units of α_2_δ3^+/+^ but failed to phase-lock for higher modulation frequencies (Fig. 3). This was also reflected by the vector strength (Fig. 8) yet even better shown by the Pearson-like (offset-corrected) cross-correlation coefficient CC_PC_ (Fig. 9). Taken together, the responses of α_2_δ3^−/−^ units were not phase-locked to the stimulus envelope at modulation frequencies ≥70 Hz.

### Neuronal α_2_δ proteins

The α_2_δ proteins α_2_δ1 – α_2_δ4 are expressed in the nervous system, muscle and endocrine organs, with α_2_δ1, α_2_δ2, and α_2_δ3 expressed in the brain (Cole et al., 2005; Ablinger et al., 2020; Geisler et al., 2021). They function as largely extracellular components of voltage-activated Ca^2+^ channels and modulate the abundance and biophysical properties of these channels (Catterall et al., 2005; Dolphin, 2013). Moreover, α_2_δ proteins may also act independently of channel function such as (1) in organizing synapses in development; (2) trafficking Ca^2+^ channels along axons; or (3) in transsynaptic coupling (Eroglu et al., 2009; Kurshan et al., 2009; Pirone et al., 2014; Fell et al., 2016; Kadurin et al., 2016; Dolphin, 2018; Ferron et al., 2018; Geisler et al., 2019, 2021; Ablinger et al., 2020; Bikbaev et al., 2020).

Lack of α_2_δ3 resulted in altered pain processing, increased acoustic startle response, altered auditory processing, cortical sensory cross-activation, anxiety-like behavior, and a volume decrease in corpus callosum (Neely et al., 2010; Pirone et al., 2014; Landmann et al., 2018; Geisler et al., 2021). Of note, the lack of α_2_δ3 in α_2_δ3^−/−^ mice is partially rescued by α_2_δ1 and α_2_δ2, as shown in a recent study on single and double knock-out mice for the brain-specific subunits α_2_δ1, α_2_δ2, and α_2_δ3 (Geisler et al., 2021).

### α_2_δ3 protein as a component of presynaptic Ca^2+^ channels in the central auditory system

The neuronal information flow along the ascending auditory pathway employs fast and reliable glutamatergic synapses with presynaptic Ca_v_2.1 Ca^2+^ channels (Lin et al., 2011) and postsynaptic AMPA receptors containing predominantly GluA3 and GluA4 subunits (Yang et al., 2011; García-Hernández et al., 2017). All three neuronal isoforms of auxiliary α_2_δ subunits of voltage-gated Ca^2+^ channel are key organizers of glutamatergic synapses and can co-assemble with any high voltage-activated Ca^2+^ channel, either presynaptic or postsynaptic or somatic (Ablinger et al., 2020; Geisler et al., 2021; Schöpf et al., 2021). Because α_2_δ3 is strongly expressed in principal neurons in the auditory pathway such as spiral ganglion neurons, neurons of the dorsal and ventral cochlear nucleus, the medial nucleus of the trapezoid body (MNTB), the ventral nucleus of the lateral lemniscus (VNLL), and in some neurons of the IC itself (Cole et al., 2005; Pirone et al., 2014; Stephani et al., 2019), it appears to play a specific role at the ultrafast Ca_v_2.1-containing synapses along the auditory pathway.

Mice deficient for α_2_δ3 showed normal function of inner and outer hair cells and nearly normal hearing thresholds (Pirone et al., 2014). However, the prominent synapse of auditory nerve terminals onto bushy cells in the anteroventral cochlear nucleus, the endbulb of Held synapse, displayed reduced evoked rates and increased latencies of postsynaptic action potentials by 0.78 ms (Pirone et al., 2014), most likely caused by smaller and malformed presynaptic terminals and reduced numbers of Ca_v_2.1 channels (Pirone et al., 2014; Stephani et al., 2019). If other synapses of auditory nerve fiber terminals had similar defects, less excitatory input would be fed into the auditory brainstem. So far, the endbulb synapse is the only central synapse studied *in vivo* in the α_2_δ3^−/−^ mouse model. If α_2_δ3 played a similar role in glutamatergic synaptogenesis and synaptic function in more centrally located auditory nuclei, the temporal processing deficits of α_2_δ3^−/−^ mice would add up. However, auditory processing in brainstem and midbrain not only involves bottom-up but also top-down signaling. Much of the exquisite extraction of timing information and phase locking was impossible without inhibitory input by interneurons at each level of brainstem and midbrain processing (Eggermont, 2015). Of note, both α_2_δ1 and α_2_δ3 subunits have been recently shown to drive the balance between excitatory and inhibitory network formation in development (Bikbaev et al., 2020). The pathomechanisms in α_2_δ3^−/−^ mice may therefore also include imbalances between excitatory and inhibitory networks, which impair precise temporal coding (Kurt et al., 2006; Bendor, 2015; Cai and Caspary, 2015) and also appear to underlie forms of ASDs (Sohal and Rubenstein, 2019).

In an auditory discrimination learning experiment, we previously found that despite the ability of α_2_δ3^−/−^ to discriminate PTs they failed to discriminate AM tones with f_m_ of either 20 or 40 Hz (Pirone et al., 2014). The results of the present study indicating more slowly responding phasic IC units in α_2_δ3^−/−^ mice at f_m_ of ≳30 Hz (see Results) are in accordance with the deficits of α_2_δ3^−/−^ mice in the auditory discrimination learning experiment (Pirone et al., 2014).

### Auditory processing and ASDs

A [C]APD in humans, either developmental or acquired, “results from impaired neural function and is characterized by poor recognition, discrimination, separation, grouping, localization, or ordering of speech sounds without peripheral hearing loss, and does not solely result from a deficit in general attention, language or other cognitive processes” (American Academy of Audiology Clinical Practice Guidelines, 2010; Bellis and Bellis, 2015; Wilson, 2019; Dillon and Cameron, 2021). Impaired information processing starting in the auditory nerve, which leads to degraded processing of AM in the IC, will ultimately deteriorate the perception of speech and other complex sounds in more central parts of the brain. Likewise, any impairment of subcortical auditory processing in mice as shown here for α_2_δ3^−/−^ mice may lead to poor discrimination of AM sounds mimicking low frequency communication calls or similar meaningful sounds (Pirone et al., 2014; Kopp-Scheinpflug and Tempel, 2015; Felix et al., 2019).

The temporal processing deficits observed in α_2_δ3^−/−^ mice, especially the reduced ability of IC units to follow stimuli with f_m_ ≥ 70 Hz in a coordinated manner, suggest that humans with loss of *Cacna2d3* gene function may experience similar temporal processing difficulties at the level of the IC. Although the hearing range differs between mice and men (2–80 vs 16–20 kHz, respectively) the range of modulation frequencies that can be processed is similar between many vertebrate species (Singh and Theunissen, 2003; Eggermont, 2015). Loss-of-function mutations in *Cacna2d3* have been identified in individuals with severe symptoms of ASDs (Iossifov et al., 2012; Girirajan et al., 2013; De Rubeis et al., 2014). ASDs are neurodevelopmental disorders characterized by various degrees of mental disability including speech problems, impaired social interaction and repetitive, stereotyped behavior (for review, see Lai et al., 2014). Children with ASD frequently show impaired auditory processing at the level of the auditory brainstem and midbrain (Marco et al., 2011; Alcántara et al., 2012; Robertson and Baron-Cohen, 2017; Scott et al., 2018; Jones et al., 2020). If subcortical processing of AM tones in some autistic children was similarly affected as described here for α_2_δ3^−/−^ mice the understanding of phonemes and speech might be degraded, leading to difficulties in speech perception, speech understanding, and language acquisition. In addition, other types of synapses outside the auditory brainstem may be affected by lack of α_2_δ3, too, which may contribute to other autism symptoms such as impaired social interaction, anxiety, and stereotyped behavior (Lai et al., 2014). Notably, autistic individuals show deficits in decoding the non-verbal emotional content of auditory information, the affective prosody (McCann and Peppé, 2003; O’Connor, 2012; Wang and Tsao, 2015; Robertson and Baron-Cohen, 2017), an activity that requires auditory feature extraction at the subcortical level (Pannese et al., 2015).

## Synthesis

Reviewing Editor: Christine Portfors, Washington State University Decisions are customarily a result of the Reviewing Editor and the peer reviewers coming together and discussing their recommendations until a consensus is reached. When revisions are invited, a fact-based synthesis statement explaining their decision and outlining what is needed to prepare a revision will be listed below. The following reviewer(s) agreed to reveal their identity: NONE. Note: If this manuscript was transferred from JNeurosci and a decision was made to accept the manuscript without peer review, a brief statement to this effect will instead be what is listed below.

Thank you for addressing the previous reviewers’ concerns.
